# Biomineralized tetramethylpyrazine-loaded PCL/gelatin nanofibrous membrane promotes vascularization and bone regeneration of rat cranium defects

**DOI:** 10.1186/s12951-023-02155-z

**Published:** 2023-11-14

**Authors:** Xiaoyu Wu, Su Ni, Ting Dai, Jingyan Li, Fang Shao, Chun Liu, Jiafeng Wang, Shijie Fan, Yadong Tan, Linxiang Zhang, Qiting Jiang, Hongbin Zhao

**Affiliations:** 1 Laboratory of 3D Printing and Regeneration Medicine, The Affiliated Changzhou Second People’s Hospital of Nanjing Medical University, Changzhou, 213164 China; 2 Changzhou Medical Center, Nanjing Medical University, Changzhou, 213164 China; 3 Orthopedic Center of Nanjing Jiangbei Hospital, Nanjiang, 210048 China

**Keywords:** Electrospinning, Nanofibers, Biomineralization, Vascularization, Bone regeneration

## Abstract

Conventional electrospinning produces nanofibers with smooth surfaces that limit biomineralization ability. To overcome this disadvantage, we fabricated a tetramethylpyrazine (TMP)-loaded matrix-mimicking biomineralization in PCL/Gelatin composite electrospun membranes with bubble-shaped nanofibrous structures. PCL/Gelatin membranes (PG), PCL/Gelatin membranes containing biomineralized hydroxyapatite (HA) (PGH), and PCL/Gelatin membranes containing biomineralized HA and loaded TMP (PGHT) were tested. In vitro results indicated that the bubble-shaped nanofibrous surface increased the surface roughness of the nanofibers and promoted mineralization. Furthermore, sustained-release TMP had an excellent drug release efficiency. Initially released vigorously, it reached stabilization at day 7, and the slow-release rate stabilized at 61.0 ± 1.8% at 28 days. All membranes revealed an intact cytoskeleton, cell viability, and superior adhesion and proliferation when stained with Ghost Pen Cyclic Peptide, CCK-8, cell adhesion, and EdU. In PGHT membranes, the osteogenic and vascularized gene expression of BMSCs and human vascular endothelial cells was significantly upregulated compared with that in other groups, indicating the PGHT membranes exhibited an effective vascularization role. Subsequently, the membranes were implanted in a rat cranium defect model for 4 and 8 weeks. Micro-CT and histological analysis results showed that the PGHT membranes had better bone regenerative patterns. Additionally, the levels of CD31 and VEGF significantly increased in the PGHT membrane compared with those in other membranes. Thus, PGHT membranes could accelerate the repair of cranium defects in vivo via HA and TMP synergistic effects.

**Graphical Abstract:**

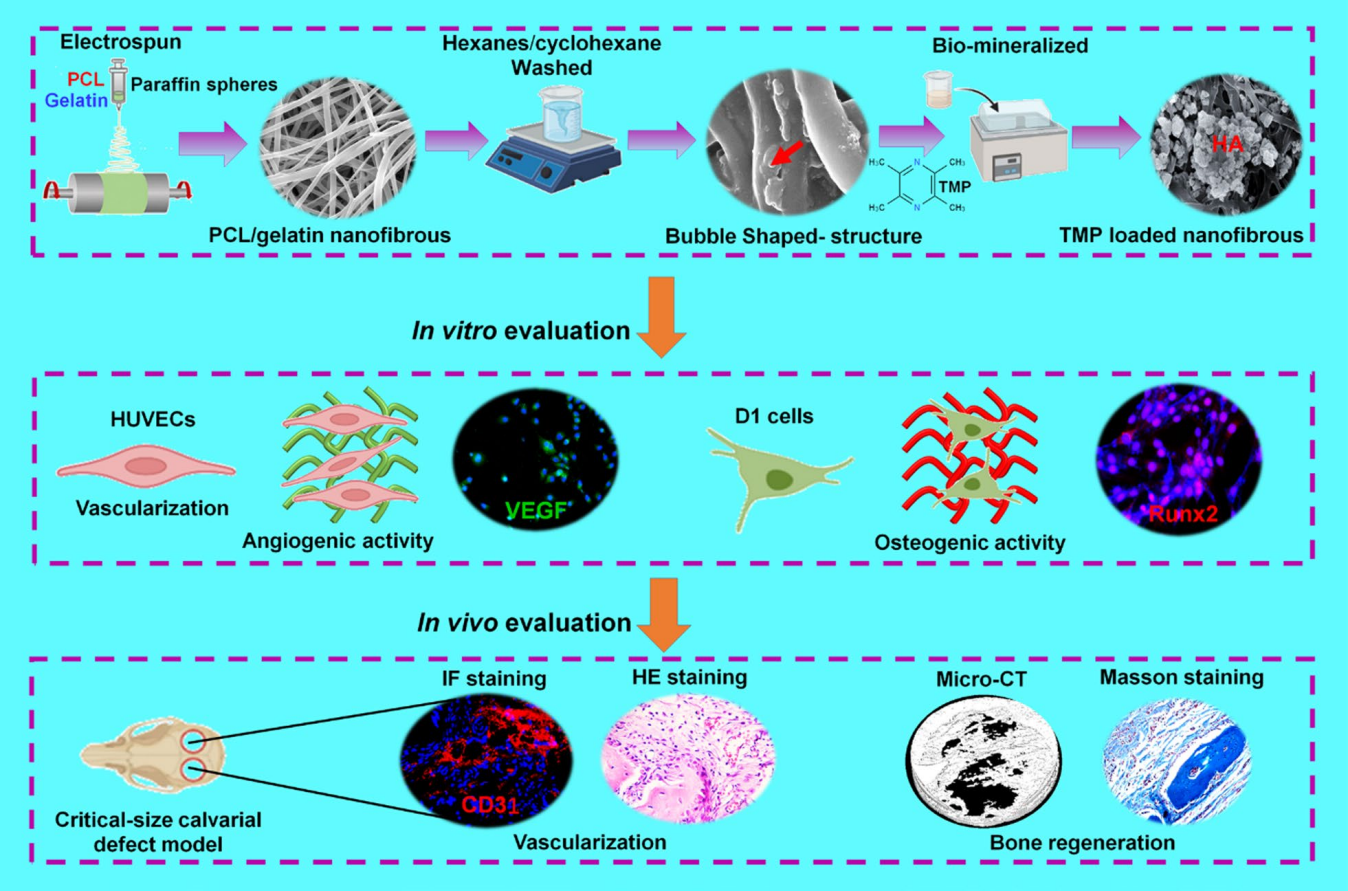

## Introduction

Electrospinning is a widely used approach for the processing of nanofibers [1–3], and various natural and synthesized components have been used to fabricate electrospun nanofibers. Synthetic polyesters, such as PCL [4], poly(lactic acid) (PLA) [5], and poly(lactic-co-glycolic acid) (PLGA) [6] have been widely used to fabricate electrospun nanofibers because of their favorable processability, chemical stability, and mechanical performance. However, these materials have several drawbacks, including low hydrophilicity and a lack of cell recognition sites that reduce cell–scaffold interactions. To address this problem, natural polymers including collagen, gelatin, silk fibroin, and chitosan have been mixed with synthetic polymers as electrospun materials to fabricate nanofibers that not only enhance the mechanical performance but also increase cell recognition sites and enhance the bioactive functions of the scaffold [7–9]. PCL has been widely used for tissue regeneration, including soft and bone tissue, and for medical delivery systems and implantable barriers [10, 11], but the slow degradation rate and hydrophobic nature of PCL limit its application as the electrospun nanofiber biomaterials. Gelatin has also become widely used due to its biological compatibility and degradability [12, 13]. However, it lacks sufficient mechanical strength and is not easy to form electrospun nanofibers. Therefore, to overcome these drawbacks, PCL and gelatin can be blended to improve the mechanical properties, hydrophobicity, and enhanced thermal stability of nanofibers compared to pure gelatin and PCL [14]. Overall, PCL/gelatin have been applied as electrospun materials to fabricate bioactive nanofiber membranes with increased mechanical performance that mimics the native extracellular matrix, which can promote the growth, proliferation, differentiation of cells, and bone formation [15, 16].

However, the smooth surface of conventional electrospun nanofibers not only decreased the biomineralization ability, but also did not allow cells to adhere and proliferate on the surface of the fibers, eventually affecting tissue formation. Recently, several approaches have been explored to overcome this disadvantage. One approach is the salt-leaching method [17, 18], where salt-generated pores on the surface of nanofiber can enhance cell adhesion because the salt increases the roughness of the electrospun fibers. However, sodium chloride can only form tiny pores on the surface of nanofibers. Furthermore, washing of NaCl crystals from electrospun sheets easily leads to the loss of nanofibers and the incomplete structure of membrane materials [19]. Wu et al. [20] reported mixing a gas-foaming and salt-leaching approach to increase the roughness of nanofibers. This unique fiber structure had a rougher surface than that of conventional nanofibers. X Jing et al. [21] studied a unique “shish kebab”–shaped scaffold. Sodium bicarbonate particles were mixed with the electrospun solution during electrospinning. Afterwards, the scaffold was immersed in a citric acid solution. Sodium bicarbonate in the material reacts with citric acid to produce carbon dioxide gas, forming crater-like structures on the surface of nanofibers. Bai et al. [22] also invented an unusually kebab-shaped scaffold. PCL nanofibers can promote the formation of crystals in the PCL domain of chitosan-polycaprolactone copolymer and finally form a beaded structure on the surface of PCL nanofibers. This shish-kebab structure of the scaffold produced increased surface roughness and therefore enabled enhanced cell attachment, viability, and proliferation.

Vascularization plays an extremely important role in bone regeneration [23, 24]. Therefore, a larger number of biomaterials or approaches have been used to induce angiogenesis. A widely used approach for angiogenesis is the application of electrospun nanofibrous membranes because of the large specific surface area of that can promote endothelial cell proliferation and angiogenesis. Furthermore, the electrospun nanofibers incorporated or conjugated bioactive molecules including growth factors (e.g., vascular endothelial growth factor, VEGF) [25] or bioactive components drugs (e.g., bioactive glasses) [26] or drugs (e.g., deferoxamine) [27] that increase angiogenesis. Yao et al. fabricated an electrospun scaffold functionalized with a hydrogen sulfide donor, which promoted HUVEC proliferation in vitro and accelerated neovascularization in vivo [28]. Du et al. fabricated a nanohydroxyapatite/coralline (nHA/coral) block coated with hVEGF scaffolds in critical size mandibular defects, which showed that the VEGF-coated scaffold promoted vascularization in the early stage of bone regeneration.

Tetramethylpyrazine (TMP, also known as ligustrazine) is one of the most primary biologically active ingredients isolated from the traditional Chinese herb ligusticum (or cnidium) and has been widely applied in treating cardiovascular diseases and ischemic neural disorders [29, 30]. Previous studies have reported that TMP may inhibit blood viscosity depression, platelet aggregation, blood vessel dilation, coronary and cerebral blood flow increase, vascular recanalization and endotheliocyte proliferation and migration promotion, and reactive oxygen species scavenging. Zhang et al*.* [31] demonstrated that TMP could enhance the migration and proliferation of brain endothelial cells. Jiang et al*.* [32] reported that TMP not only inhibited steroid-induced osteonecrosis but also increased bone mineral density (BMD). Moreover, TMP remarkably increased the vascularization of the femoral heads. Therefore, we hypothesized that using the biomineralization method to load TMP on the surface of electrospun nanomaterials, and through the rapid release of TMP drugs to promote the vascularization of new tissue and accelerate bone regeneration and repair, may be a new strategy and approaches.

Herein, PCL and gelatin were mixed with paraffin spheres to fabricate bubble-shaped nanofibrous TMP-loaded matrix-mimicking biomineralized electrospun membranes. Our hypothesis is that the bubble-like shapes on the nanofibrous surface increase the surface roughness and specific surface area, which can improve biomineralization ability and produce a matrix that mimics the mineralized microenvironment. Additionally, loaded TMP was released from the mineralized matrix to enhance vascularization and new bone formation. We characterized the bubble-like structures and tested their chemical, mechanical, and biological properties. Cell attachment, viability, and proliferation were examined in vitro. We evaluated the in vivo vascularization and new bone formation induced by the membranes. Our results indicated that the active roles of the bubble-shaped nanofibrous TMP-loaded matrix-mimicking mineralized membrane in increasing cell attachment, viability, differentiation and enhancing the vascularization and new bone formation. This novel membrane is an ideal biomaterial for bone regeneration.

## Materials and methods

### Fabrication of PCL/Gelatin electrospinning nanomembranes

PCL (80 kDa, Aladdin Reagents Ltd., China) was dissolved in hexafluoroisopropanol solution (HFP; Sigma-Aldrich Inc., USA) with gentle stirring for at least 24 h to obtain a 10% w/v solution. Gelatin (300 Bloom, Aladdin Chemical Inc., China) was dissolved in HFP to obtain 10% w/v gelatin homogenous solution. Then, the 10% w/v PCL solution was added to the 10% w/v gelatin solution until it was mixed completely at room temperature. Finally, 0.5 g of paraffin sphere particles (100–200 µm) were dispersed into the PCL/Gelatin electrospun solution by stirring overnight at room temperature for at least 24 h before use. The PCL/Gelatin solutions were subsequently loaded into a 10 mL plastic syringe with two 20-gage needles and installed in the propulsion system. The PCL/Gelatin fibers were obtained using the electrospinning machine (YFSP-T Tianjin Yun Fan Tech. Ltd.) at 16 kV voltage, 12 cm collection distance, and a rate of 2 mL/h. The average thickness of nanofiber sheets was 2–3 mm. When completed, the PCL/Gelatin sheets were washed using deionized (DI) water overnight and vacuum freezing (− 40 °C) for 48 h. The PCL/Gelatin membranes were cut into 20 × 20 mm (length × width). All samples were immersed in hexane at room temperature overnight with rotation, then washed with cyclohexane at least 12 h at room temperature, and subsequently immersed in absolute ethyl alcohol for 24 h. Finally, the samples were vacuumed at 25 °C overnight to remove all residual cyclohexane and then stored at room temperature until further use in the desiccator.

### Fabrication of biomineralized TMP-loaded PCL/Gelatin nanofibrous membrane

Mineralization of PCL/Gelatin nano-membranes was performed as previously reported [33]. Briefly, the PCL/Gelatin membranes were immersed in the nucleation solution (0.6 M NaCl, 0.02 M CaCl_2_, 0.05 M NaHPO_4_·2H_2_O, 0.02 M NaHCO_3_, 0.075 M MgCl_2_·6H_2_O) for 24 h at 120 rpm and 37 °C. The PCL/Gelatin nanofiber sheets were washed with DI water, frozen, and then freeze-dried overnight. The freeze-dried membranes were then soaked in propagation solution (10 M HCl (2.5 mL), 0.04 M CaCl_2_, 0.14 M NaCl, 0.003 M NaHPO_4_·2H_2_O, pH = 7.4), meanwhile, TMP (4 g, maximum solubility) was added to the 1 L of propagation solution until this dissolved completely at 37 °C. The membranes were then soaked in propagation solution (with TMP fabrication biomineralized TMP-loaded PCL/Gelatin nanofibrous membrane; without TMP fabrication biomineralization of PCL/Gelatin membrane) and shaken at 37 °C for 72 h. Finally, the membranes were washed with DI water and frozen at − 20 °C for 12 h. Followed by freeze-drying at − 40 °C for 24 h, and were kept at − 20 °C until use. PCL/Gelatin membrane was named PG, biomineralization of PCL/Gelatin membrane was named PGH, biomineralization loaded TMP PCL/Gelatin membrane was named PGHT.

### Determination of drug loading rate of membranes

Dissolve 100 mg of PGHT membranes in 10 mL of acetonitrile and vortex mix for 10 min before allowing to stand for 20 min. After the drug on the membranes is fully dissolved and released, centrifuge the supernatant and measure the drug concentration using a UV spectrophotometer (Shimadzu, Japan) with an ultraviolet wavelength of 302 nm. Calculate the average drug loading rate of the membranes after repeating three experiments. The drug loading rate were calculated according to the following formula [34].

$$\text{Drug loading rate }({\%})=\frac{W1}{W2}\times 100\%$$

where W1 is drug weight in membranes, W2 is total membranes weight.

### TMP cumulative-release measurement

100 mg PGHT membranes were placed in a 50 mL centrifuge tube. The membranes were immersed in 50 mL of phosphate-buffered saline (PBS; 0.2 M) and shaken at 37 °C. Supernatant (1 mL) was removed from PBS at different time points (1, 2, 3, 4, 7, 14, 21, 28, and 35 days) and then an equal volume fresh PBS was used to replenish the solution in the centrifuge tube. The supernatant (1 mL) was used for analysis of the drug concentration with an ultraviolet spectrophotometer at 302 nm (Shimadzu, Japan). The cumulative release of TMP was calculated according to the following formula [35].

$$ {\text{Cumulative}}\,\,{\text{release}}\,{\text{(\% )}} = \frac{{{\text{V1Cn}} + v2\sum {C(N - 1)} }}{{\text{W}}} \times 100 $$

where Cn is the concentration of TMP in the supernatant at predetermined time points. V1 is the total volume of PBS (50 mL), and V2 is the supernatant-volume time points (1 mL). W (6.12 µg) is the initial amount of TMP loaded in the membranes.

### Physical and chemical characterization

#### Scanning electron microscopy

Membranes were cut into a square of 20 × 20 mm (length × width). The morphology and topography of the nanofiber membranes were characterized using scanning electron microscopy (SEM). Samples were fixed on a copper column and then coated with platinum powder for 360 s. SEM analysis was performed at 12 kV. The average diameter of the nanofibers was measured using ImageJ (National Institutes of Health, USA).

### Fourier-transform infrared spectroscopy

The functional chemical groups of the membranes were evaluated using Fourier-transform infrared (FTIR) spectroscopy (Avatar 360, Nicolet, USA). The samples were sectioned into a square of 20 × 20 mm (length × width), and the spectra of each sample were recorded with a resolution of 4 cm^−1^ (64 scans) between 400 to 4000 cm^−1^.

### Porosity of membranes

Membranes were soaked in ethanol (10 mL) for 10 min, and then the weight was measured before (W1) and after immersion (W2). Samples were removed from ethanol, and the weight of residual ethanol was measured (W3). *ρ* is the ethanol density. The porosity of the membranes was determined using the following formula [36]:

$${\text{Porosity}} \, (\%)=\frac{{(W1-W3)/\rho }}{{(W2-W3)/\rho }} \times 100{\%}$$

### Water contact angle measurements

The static contact angle of all nanofiber sheets was measured using water contact angle analysis (Dataphysics, Germany). Samples (1 × 1 cm, length × width) (n = 3) were placed on the testing plate and kept smooth. Afterward, the water contact angle was tested by slowly dropping 0.01 mL of DI water on the surface of the nanofiber sheets, and then recording the angle value every 10 s by the video monitor.

### Air permeability of the membranes

The air permeability of the various nanofibers sheets was assessed using the ASTM E96/E96M-15 Standard Test Method. The test membrane (n = 3) was sealed to the open mouth of the measuring bottle filled with 10 mL of DI water. After that, the bottle was kept in an incubator for 3 days at 37 °C, and the weight change measured periodically. The air permeability rate of the membranes was calculated according to the following formula [41]:

$$ \text{Air permeability rate} (\%) =\frac{ \text{Mass lost}}{T \times Area} \times 100\%$$

where air permeability rate is the water vapor transmission rate through the membrane (g water. m^−2^ day^−1^). Mass lost is the mass loss of water daily, Area is the area (m^2^) of the membrane. T [day] is the accumulation time.

### Mechanical properties of membranes

The tensile properties of the nanofibers sheet were tested using a universal mechanical testing machine (3367; Instron, Norwood, MA). The membranes were trimmed to a section measuring 4 × 1 cm^2^ (length × width) and tested at room temperature (n = 3). The initial spacing between the claws was 20 mm with a load cell of 30 N, and the stretching speed was 3 mm min^−1^. The stress–strain curve was drawn according to the testing data.

### Cell culture

Mouse bone marrow progenitor cell line (D1 cells) were purchased from ATCC (USA, ATCCCRL-12424), and were cultured in Dulbecco’s Modified Eagle Medium (DMEM; Gibco, USA) cell complete culture medium containing 10% fetal bovine serum and 1% penicillin/streptomycin at 37 °C and 5% CO_2_. The medium was changed twice a week.

### Cell proliferation

D1 cells (2 × 10^5^ cells/sample) were seeded onto the prewetted membranes (20 × 20 × 0.3 mm, length × width × thickness) in a 24-well plate. On days 1, 3, and 5 after co-culturing, the membranes were removed, washed, and transferred to a new 24-well plate, with 300 µL of fresh medium containing CCK-8 reagent (ratio 1:10) (Dojindo Molecular Technologies, Inc.) and then incubated at 37 °C for 2 h. Subsequently, suspensions (100 µL) were collected from each well, and placed in a 96-well plate. Finally, the absorbance was measured at 450 nm using an ultraviolet spectrometer (Shimadzu, Japan).

### EdU incorporation assay

Click-ITTM 5-Ethynyl-2′-deoxyuridine (EdU) Alexa Fluor488 Kit (Life Technologies) was employed to assess the cell proliferation. D1 cells were co-cultured with the PG, PGH, and PGHT membranes in 24-well plates for 24 h and were then incubated with the complete culture medium (containing 50 µM EdU) for 2 h. The cells were fixed with 4% paraformaldehyde and permeabilized with 0.5% Triton X-100, and the cells were then stained in a solution of 10 µM DAPI for 10 min. After staining, fluorescent images were captured using a fluorescence microscope (Zeiss, Oberkochen, Germany) and then EdU^+^ cells were computed and counted.

### Live/dead staining

D1 cells (1 × 10^5^ cells/sample) were seeded on the different membranes (20 × 20 × 0.3, length × width × thickness) in a 24-well plate and cultured in DMEM complete medium. On day 7, cells were washed with PBS (0.2 M, pH = 7.4) three times, and incubated with 0.6 mL of live/dead staining solution (Invitrogen) in the dark at room temperature for 30 min. After washing with PBS, samples were captured and distinguished live/dead cells using a fluorescence microscope (Zeiss, Oberkochen, Germany). The ratio of live/dead cells was calculated according to the image of staining.

### Alizarin Red S and ALP staining

Alizarin Red staining: D1 cells (2 × 10^4^ cells/membrane) were seeded on the different membranes (20 × 20 × 0.3 mm) in a 24-well plate and cultured in DMEM complete medium for 24 h; the medium was then removed, osteogenic medium containing β-glycerophosphate (5 mM), dexamethasone (100 nM), and ascorbic acid diphosphate (50 µM) was added and continuously cultured for 14 days. On day 14 post-cultivation, the samples were washed twice in PBS, and were subsequently stained in Alizarin Red S (0.1%) solution. After washing twice with DI water, the absorbance was read at 405 nm using a microplate reader.

To assess the bone induction ability of membranes, D1 cells (2.0 × 10^5^ cells/membrane) were seeded on the different membranes (20 × 20 × 0.3 mm) in 24-well plates. On days 1 and 7 of osteogenic induction, cells were fixed with acetone for 10 min at 4 °C. An ALP staining kit (Beyotime, Suzhou, China) was performed to analyze the bone induction ability.

### Real-time qPCR analysis

D1 cells were seeded on the different membranes (20 × 20 × 0.3 mm) with a density of 2.0 × 10^6^ cells/membrane in a 12-well plate and cultured in DMEM complete medium on days 1, 3, and 7. The total RNA was collected using the NucleoZol kit (Macherey–Nagel, Germany) according to the manufacturer’s protocols. Total RNA was converted to cDNA using the HiScript II Q RT SuperMix reagent Kit (Vazyme, China). The Taq-TM qPCR kit (Vazyme, China) was performed for qPCR. The target gene expression was quantified using the 2^−ΔΔCt^ method. Primers are described in Table 1. qPCR reactions were tested in triplicate for all targets and the housekeeping gene *GAPDH* as the housekeeping gene.

**Table 1:** Real-time qPCR primer sequences

Gene	Primer	Primer sequence
GAPDH	Forward	5′—TTCACCACCTTCTTGATGTC-3ʹ
	Reverse	5′—CACCACCAACTGCTTAGC-3ʹ
ALP	Forward	5′—ATGGCCTGGTCCATCTCCAC-3ʹ
	Reverse	5′—GCAGTATGAATTGAATCGGAACAAC-3ʹ
OPN	Forward	5′—TATAGGATCTGGGTGCAGGCTGTA-3ʹ
	Reverse	5′—TACGACCATGAGATTGGCAGTGA-3ʹ
Runx-2	Forward	5′—GGCTCACGTCGCTCATCTT-3ʹ
	Reverse	5′—CTGCAAGCAGTATTTACAACAGAGG-3ʹ
Osterix	Forward	5′—GCCAGATGGAAGCTGTGAAGA-3ʹ
	Reverse	5′—AGGCCTTTGCCAGTGCCTA-3ʹ

### Expression of angiogenesis-related gene on the different membranes

HUVECs (8 × 10^5^ cells per membrane) were co-cultured with the different membranes on days 1 and 3. Total RNA collection, cDNA reverse transcription, and real-time qPCR analysis were performed to assess the expression of angiogenesis-related marker genes, such as hypoxia-inducible factor (*HIF1A*), fibroblast growth factor (*FGF*), and platelet endothelial cell adhesion molecule-1 (*CD31*). Real-time qPCR primer sequences are described in Table 2. Data were quantified using the 2^−ΔΔCt^ relative quantification method for the target gene expression.

**Table 2:** Real-time qPCR primer sequences

Gene	Primer	Primer sequence
GAPDH	Forward	5′—CACCACCAACTGCTTAGC-3ʹ
	Reverse	5′—TTCACCACCTTCTTGATGTC-3ʹ
HIF 1A	Forward	5′—GAACGTCGAAAAGAAAAGTCTCG-3ʹ
	Reverse	5′—CCTTATCAAGATGCGAACTCACA-3ʹ
FGF	Forward	5′—GAGCGACCCACACGTCAAACTAC-3ʹ
	Reverse	5′—CAGCCGTCCATCTTCCTTCATAGC-3ʹ
CD31	Forward	5′—AGCCAGCAGTATGAGGACCAGTC-3ʹ
	Reverse	5′—TCCAATGACAACCACCGCAATGAG-3ʹ

### Immunofluorescence assays

Seven days after being co-cultured with the different membranes, cells were fixed in ice-cold alcohol (70% v/w) at 4 °C overnight, permeabilized using 0.1% TritonX-100, and deactivated the endogenous peroxidase with 3% H_2_O_2_. Nonspecific antigens were then blocked with 5% goat serum for 30 min at room temperature, and the cells incubated with the primary anti-Runx2 antibodies (1:200; Abcam) overnight at 4 °C, followed by Cy3-labeled secondary antibodies goat anti-mouse IgG (Abcam, USA) at 37 °C for 30 min. After that, 3 µM DAPI (Sigma, USA) for nucleus staining was added for 10 min. Cells were subsequently imaged using a fluorescence microscope (Zeiss, Oberkochen, Germany).

### In vivo animal experiments

Sixty SD male rats (weights 240–260 g) were randomized into four groups (n = 15) to evaluate in vivo bone regeneration: (1) No material was implanted as the control group; (2) rats were implanted with the PG membrane; (3) with the PGH membrane; and (4) with the PGAT membrane. The cranium defect models were generated with a 5 mm diameter and 0.5 mm depth. The rats were anesthetized with pentobarbital (100 mg/kg, intraperitoneal). Two symmetrical circular cranium defects were made using a trephine bur (5 mm diameter). Subsequently, the different membranes (5 mm diameter and 0.3 mm thickness) were implanted in the circular cranium defects, respectively. At weeks 4 and 8 post-operation, the rats were euthanized, and cranium samples were removed and fixed with 4% paraformaldehyde buffer for 3 days before further experiments. All animal experiments were performed in strict accordance with the Affiliated Changzhou No. 2 People’s Hospital of Nanjing Medical University animal ethics committee guidelines.

### Micro-CT analysis

Micro-CT Analyzer (1.8.0.2, SkyScan) was used to analyze the microarchitecture of the rat cranium defects at weeks 4 and 8 post-surgery. The SD rats were euthanized, the cranium was fixed using 10% formaldehyde. Images were scanned at 45 kV and 22 µA with a scan thickness of 18 µm, and then three-dimensional reconstruction image was analyzed using NRecon (1.4.4, SkyScan) software.

### Histological staining

The rat craniums were demineralized with EDTA (10% w/v) after micro-CT examination, embedded in paraffin, and then sectioned into 5 μm slices. Samples were stained with hematoxylin and eosin (H&E) and Masson’s trichrome stains according to the protocol. The images of staining were captured under light microscopy (Zeiss, Oberkochen, Germany).

### Immunohistochemistry

Immunohistochemistry was used to evaluate the expression of osteogenesis-related proteins in paraffin sections. Tissue sections were deparaffinized and then immersed in an antigen repair solution (0.5 g of trypsin, 1 g of anhydrous calcium chloride, and pH = 7.8) for target antigen retrieval at 37 °C for 1 h. After washing three times with DI water, tissue sections were soaked in 3% H_2_O_2_ solution for 10 min to inhibit endogenous peroxidase activity, followed by blocking the specific antigens with 5% BSA solution for 30 min at room temperature, and then incubated with the primary anti-Runx2 (1:100, Santa Cruz), anti-OCN (1:200, Abcam), anti*-*OPN (1:200, Santa Cruz), and anti-VEGF (1:100, Abcam) antibodies overnight at 4 °C. The following day, samples were incubated with (horseradish peroxidase)-labeled rabbit anti-mouse or goat anti-rabbit secondary antibodies at 37 °C for 30 min, and subsequently SABC incubated at 37 °C for 30 min. Finally, the slides were counterstained with DAB staining at room temperature for 5–10 min. Images were captured using an optical microscope (Zeiss, Oberkochen, Germany). Furthermore, immunofluorescence staining of CD31 (anti-CD31 antibody, Abcam) and H&E staining were performed on the samples to confirm levels of neovascularization levels in vivo. Image-Pro Plus 6.0 software (Media Cybernetics, USA) was used to analyze the expression of proteins. The data show the average optical density (IOD/area).

### Statistical analysis

Data are reported as the mean ± SD (standard deviation). Statistical analysis was conducted using One-way analysis of variance followed by an LSD post hoc test; a *p*-value < 0.05 was considered statistically significant.

## Results

### Physical and chemical characteristics

#### Morphological characteristics of nanofibers

**Fig. 1:**
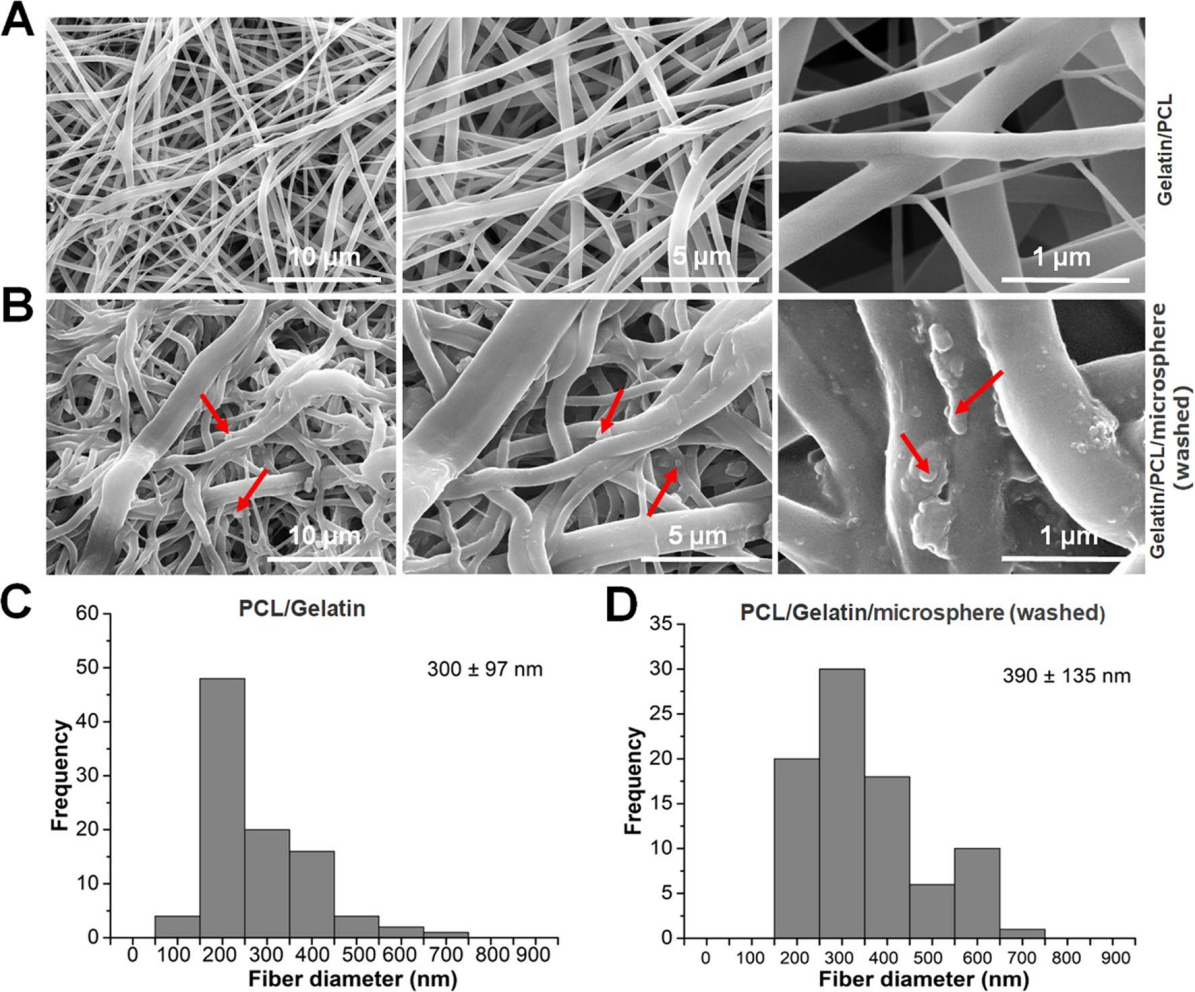
SEM graphs of PCL/Gelatin nanofibers. **A** PCL/Gelatin/paraffin sphere membrane. **B** PCL/Gelatin/paraffin sphere membrane was washed with hexane and cyclohexane. **C** Nanofiber diameter of PCL/Gelatin/paraffin sphere membrane. **D** Nanofiber diameter of PCL/gelatin/paraffin sphere membrane was washed with hexanes and cyclohexane. Red arrow: bubble shape

The SEM images of the PCL/Gelatin/paraffin sphere membrane morphology are shown in Fig. 1A. The nanofiber displayed specific micro/nanoscale structural features. Interestingly, the surface of the PCL/gelatin/paraffin membrane nanofiber that was washed with hexanes and cyclohexane exhibited the bubble-shaped structural features (Fig. 1B). The average fiber diameter of the electrospun PCL/Gelatin/paraffin spheres membrane was 300 ± 97 nm (Fig. 1C). However, as shown in Fig. 1D, by washing away the paraffin spheres, the average fiber diameter of the PG membrane increased to 390 ± 135 nm, with the formation of the bubble-like shapes on the nanofiber structure. Furthermore, this also increased the surface area and roughness of the nanofiber.

#### Characterization of biomineralized nanofiber membranes

**Fig. 2:**
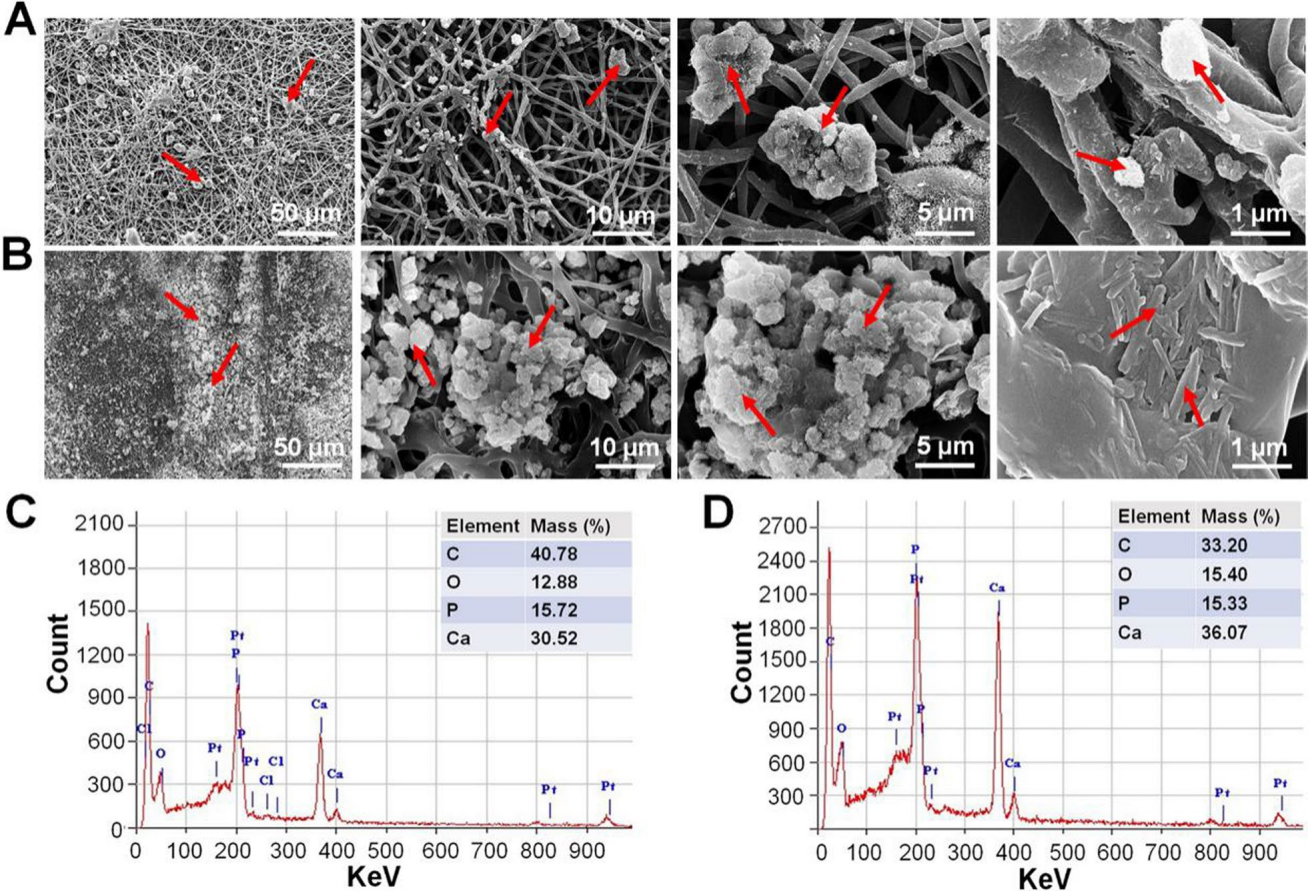
Characterization of the biomineralized PCL/Gelatin and PCL/Gelatin/paraffin sphere membranes. **A** SEM images of the biomineralized PCL membranes. **B** SEM images of the biomineralized PCL/Gelatin/paraffin spheres membranes that were washed with hexane and cyclohexane. **C** EDX analysis for the biomineralized PG membranes. **D** EDX analysis of the biomineralized PCL/Gelatin/paraffin sphere membranes that were washed with hexane and cyclohexane. Red arrow: calcium phosphate deposition

The characterization of the biomineralized PCL/Gelatin and PCL/Gelatin/paraffin sphere membranes that were washed with hexanes and cyclohexane is shown in Fig. 2. SEM images showed the formation of calcium phosphate deposits on the PG membrane surface. The mineralized quantities in the PCL/Gelatin/paraffin spheres membranes were significantly increased compared with that in the PG membranes (Fig. 2A, B). Under higher magnification (1 µm), the mineralized PCL/Gelatin/paraffin sphere membranes in this study exhibited the needle-like mineral precipitates deposited on the surface of the PG membrane (Fig. 2B). EDX spectroscopy results showed that P and Ca elements were present in the mineral precipitates (Fig. 2C, D). PG membranes contained Ca (30%) and P (15%), whereas PGH membranes contained 36% Ca and 15% P, indicating the presence of Ca and P on the surface of membranes due to HA particle formation in the mineral precipitates.

**Fig. 3:**
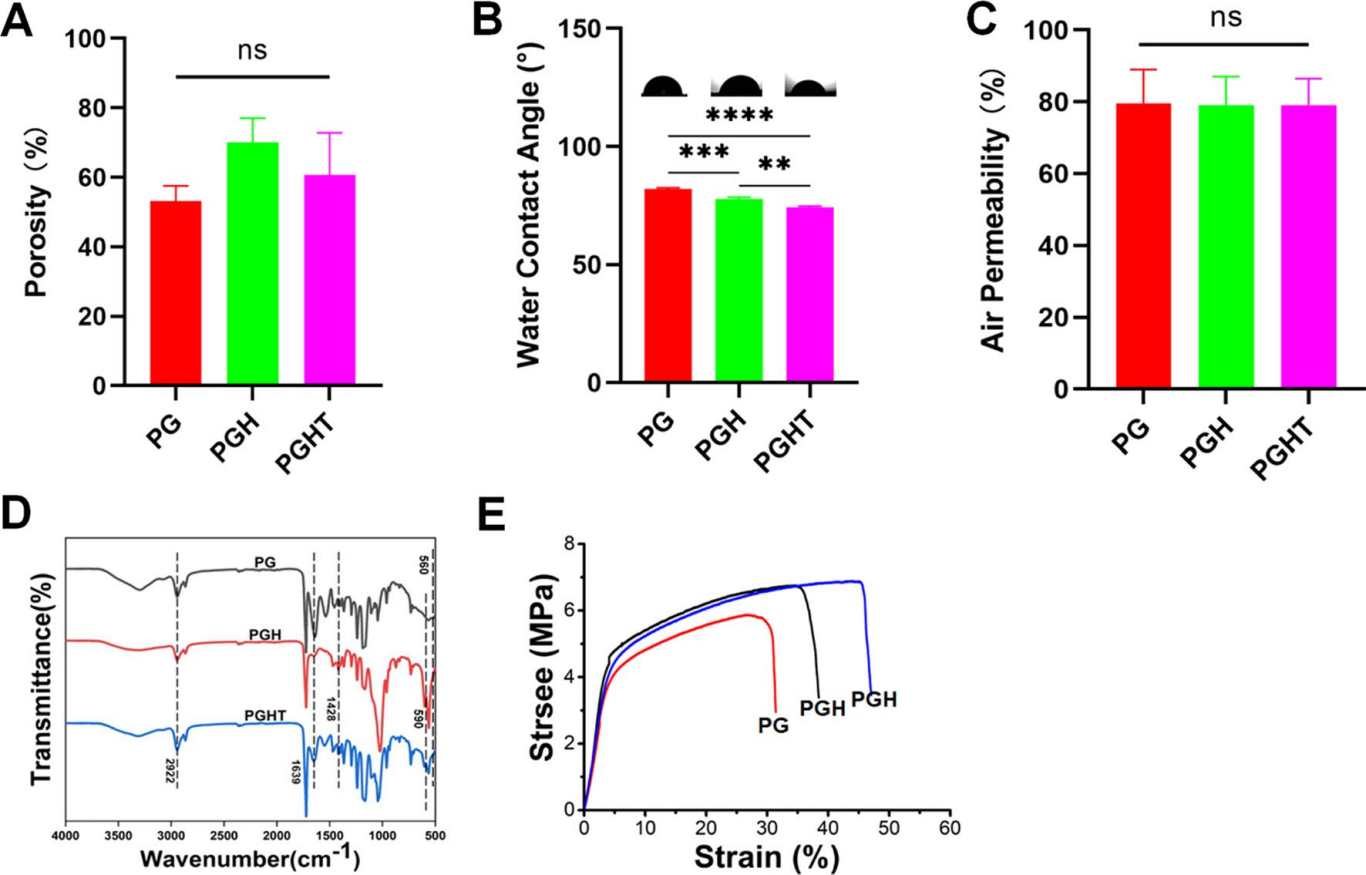
Characterization of the different membranes. **A** Porosity. **B** Water contact angle. **C** Air permeability. **D** FTIR spectra. **E** Stress–strain curve of the PG and PGH membranes. (n = 3). *p < 0.05; ** *p* < 0.01

The porosity of the different membranes is shown in Fig. 3A, and no significant difference in porosity was observed among the membranes. The water contact angle of the PGHT membrane significantly deceased in comparison with that of the PG and PGH membranes, and was lower in the PGH membrane than that in the PG membrane, due to the membranes were biomineralized. The increase in the hydrophilicity of the membranes caused by the biomineralized HA can enhance the absorption of water (Fig. 3B). Figure 3C showed that the in vitro air permeability of the different membranes did not significantly differ among the membranes, indicating the biomineralized membranes did not affect the air permeability.

The FTIR analysis of the different membranes is shown in Fig. 3D. The absorption peaks at 2922 and 1639 cm^−1^ were acylamines in gelatin, and produced by coupling N–H stretching and N–H bending vibration with C–N stretching vibration. The peal at 1428 cm^−1^ corresponds to the absorption peak of carbonate in hydroxyapatite. Additionally, notably, the peaks at 590 and 560 cm^−1^ represent the P–O band, indicating nano-HA in the membranes [37]. Analysis of the FTIR spectral profile confirmed that the successful biomineralized nano-HA had been formed in the PG membranes.

Figure 3E depict the mechanical behavior of the PG and PGH membranes, respectively. The average tensile strength and the average elongation at break of the PG membrane were 5.59 ± 0.24 MPa and 27.72 ± 3.35 mm, respectively. By contrast, the average tensile strength and the average elongation at break of the PGH membranes were 6.51 ± 0.56 MPa and 40.30 ± 4.64 mm, respectively (Fig. 3E). In comparison with those of the PG membrane, the average tensile strength in the PGH membrane was increased. This result indicated that the biomineralized PG membranes formed nano-ATP can enhance the mechanical strength of the membrane materials because of the formation of HA on the nanofiber membrane surface.

#### The drug loading rate and drug release rate in vitro

**Fig. 4:**
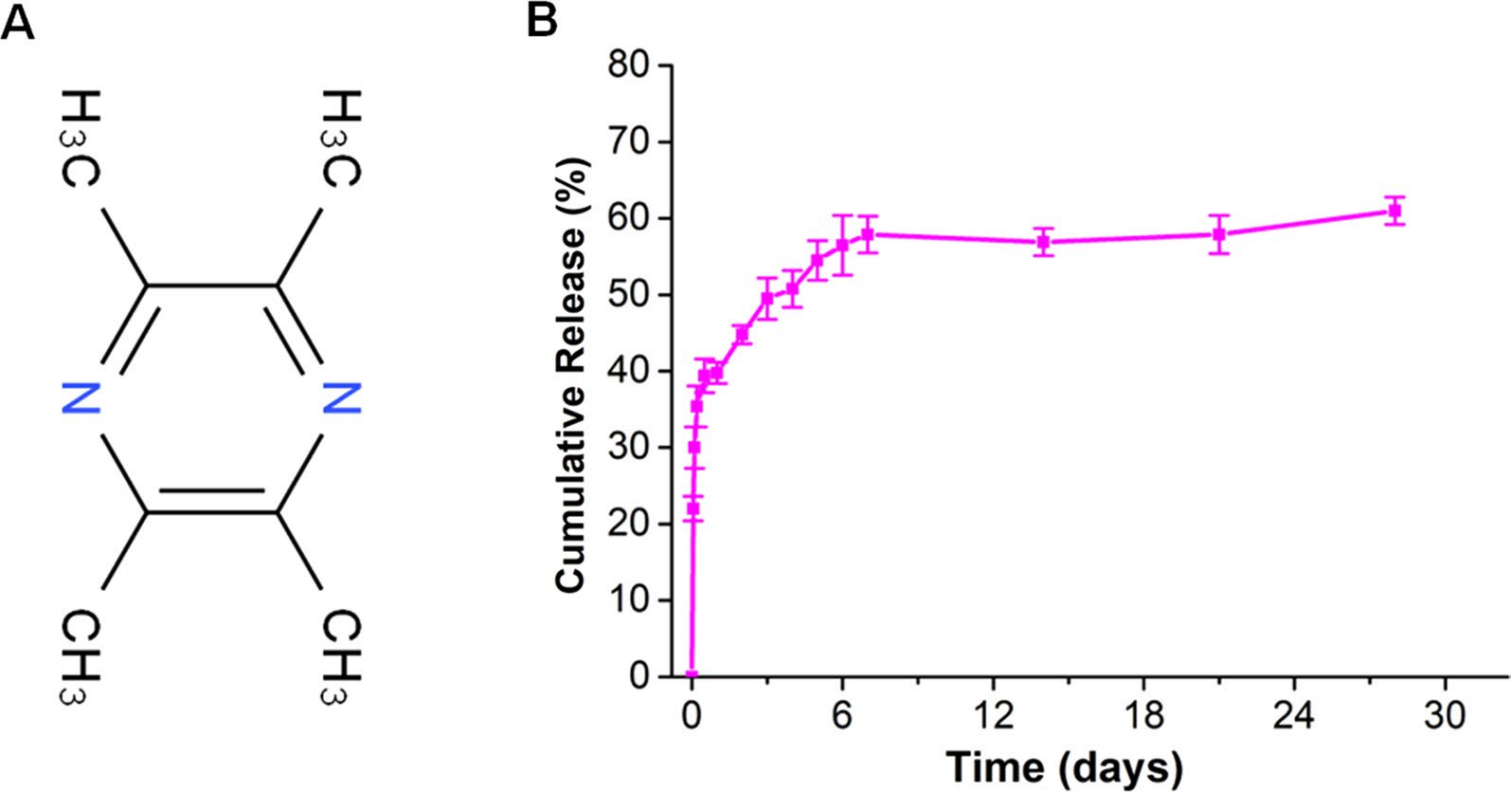
The structural formula of TMP and release profile of TMP from the PGHT nanofibers. **A** Structural formula of TMP. **B** Cumulative-release profile of TMP (n = 3)

The drug loading rate was (6.80 ± 1.57)%. Figure 4 shows the structural formula of TMP and the in vitro cumulative-release profiles of TMP from the PGHT nanofiber membrane for 28 days. Based on the cumulative-release profile exhibited, PGHT nanofibers had two stages of TMP release. One is the early burst-release of 39.8 ± 1.4% (337 ng) TMP within 2 days. The other is the slow-sustained release from 6 to 28 days before leveling off; the release of TMP increased gradually within 7 days. The cumulative release of TMP was 61.0 ± 1.8% (579 ng) at day 28 (Fig. 4B), indicating the PGHT membrane had an excellent drug release efficiency.

#### Bioactivity and biocompatibility of membranes

**Fig. 5:**
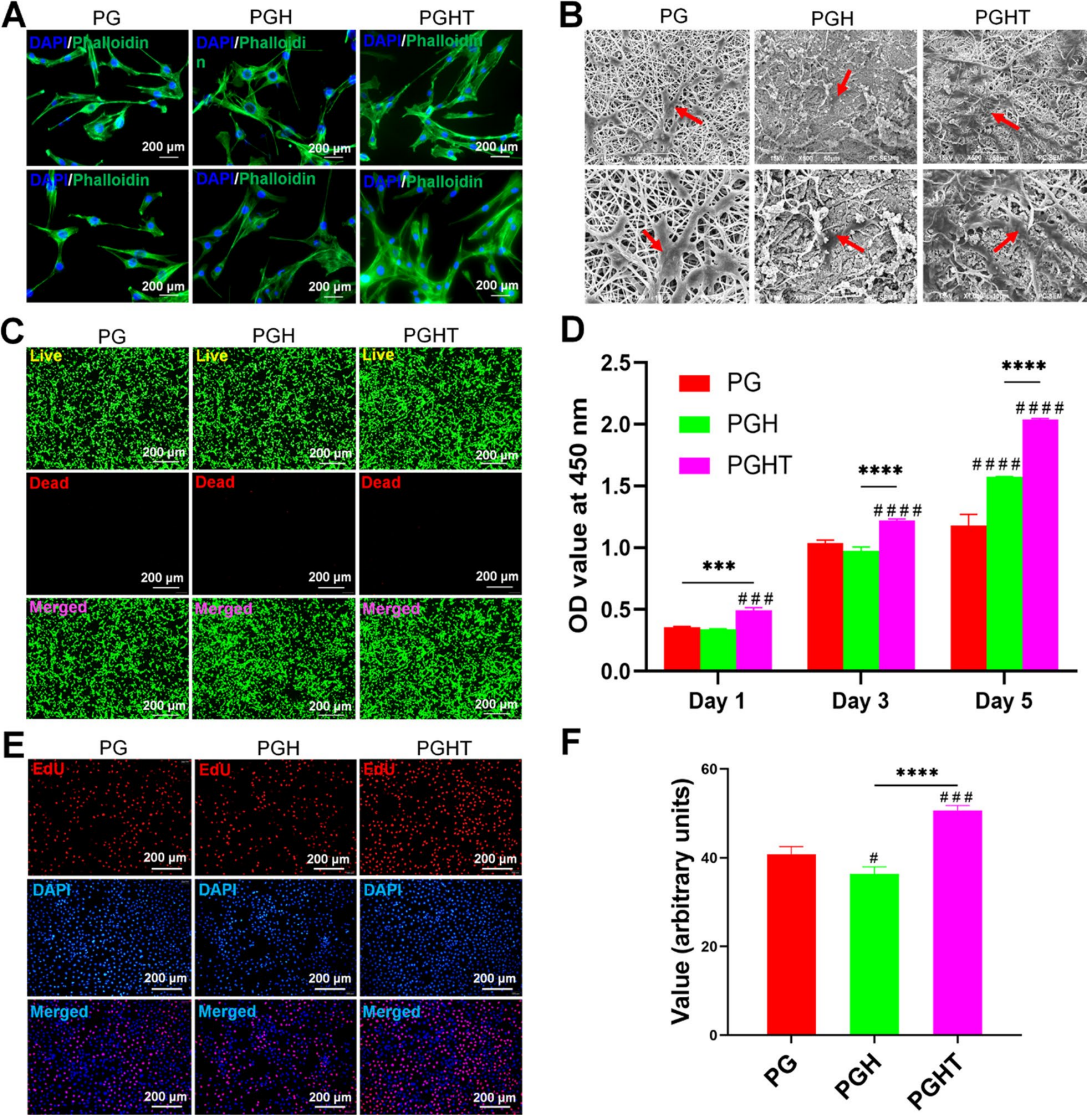
In vitro biocompatibility of membranes. **A** Immunofluorescence staining images (blue, nuclei stained with DAPI; green, cellular F-actin stained with FITC-phalloidin) of D1 cells on the different membranes on day 7. **B** SEM images of D1 cells on the surfaces of the different membranes on day 7. Red arrow: cells. **C** Live/dead staining of D1 cells expanded on the different membranes on day 7 (red, dead cells; green, live cells). **D** CCK-8 assay to determine the proliferation of D1 cells on the different membranes on days 1, 3, and 7 (n = 3). **E** Images of EdU staining of D1 cells on the different membranes on day 7 (red, EdU^+^ cells; blue, cell nuclei). **F** Semiquantitative analysis of EdU^+^ staining of cells. *** p < 0.01; **** p < 0.001 vs the PG membrane; ^###^ p < 0.01; ^####^ p < 0.001 vs the PGH membrane

To evaluate the ability of cells adherence and proliferation on the various nanofiber membranes, we employed live/dead immunofluorescence staining together with the CCK-8 assay and SEM to evaluate the bioactivity and biocompatibility of membranes. The cell adhesion behavior of cells on the membrane surface on day 7 was assessed via F-actin cytoskeleton staining using rhodamine phalloidin. The cells on the PGH and PGHT membranes exhibited better adherence (Fig. 5A); furthermore, the actin fibers were longer and straighter than those of the PG membrane. Additionally, SEM was used to evaluate the adhesion of the cells on the surface of the nanofiber membranes after seeding on day 3. This showed that the cells were firmly attached to the surface of membrane, and their abundance was significantly increased in the PGHT membrane (Fig. 5B). As shown in Fig. 5C, live/dead staining demonstrated that the vast majority of cells survived on the different membranes on day 7, with no significant difference in cell viability between the membranes. These results indicated that all membranes, especially the PGHT membrane, were suitable for cell adhesion, migration, and growth. Cell proliferation was enhanced by the PGHT membrane. The CCK-8 assay confirmed that the proliferation of cells did not differ significantly after culturing on the different membranes for 1 day. However, by days 3, and 5, the cell proliferation was markedly increased on the PGH and PGHT membranes. Notably, the PGHT membrane exhibited more proliferative effects on days 3 and 5 than that of the PG and PGH membranes did (Fig. 5D). Significantly, the PGHT membrane contained more EdU^+^ staining cells than that of the PG and PGH membranes did (Fig. 5E, F), indicating the PGHT membrane promoted cell proliferation. This result is consistent with that of the CCK-8 assay.

#### In vitro BMSC osteogenic induction assays

**Fig. 6:**
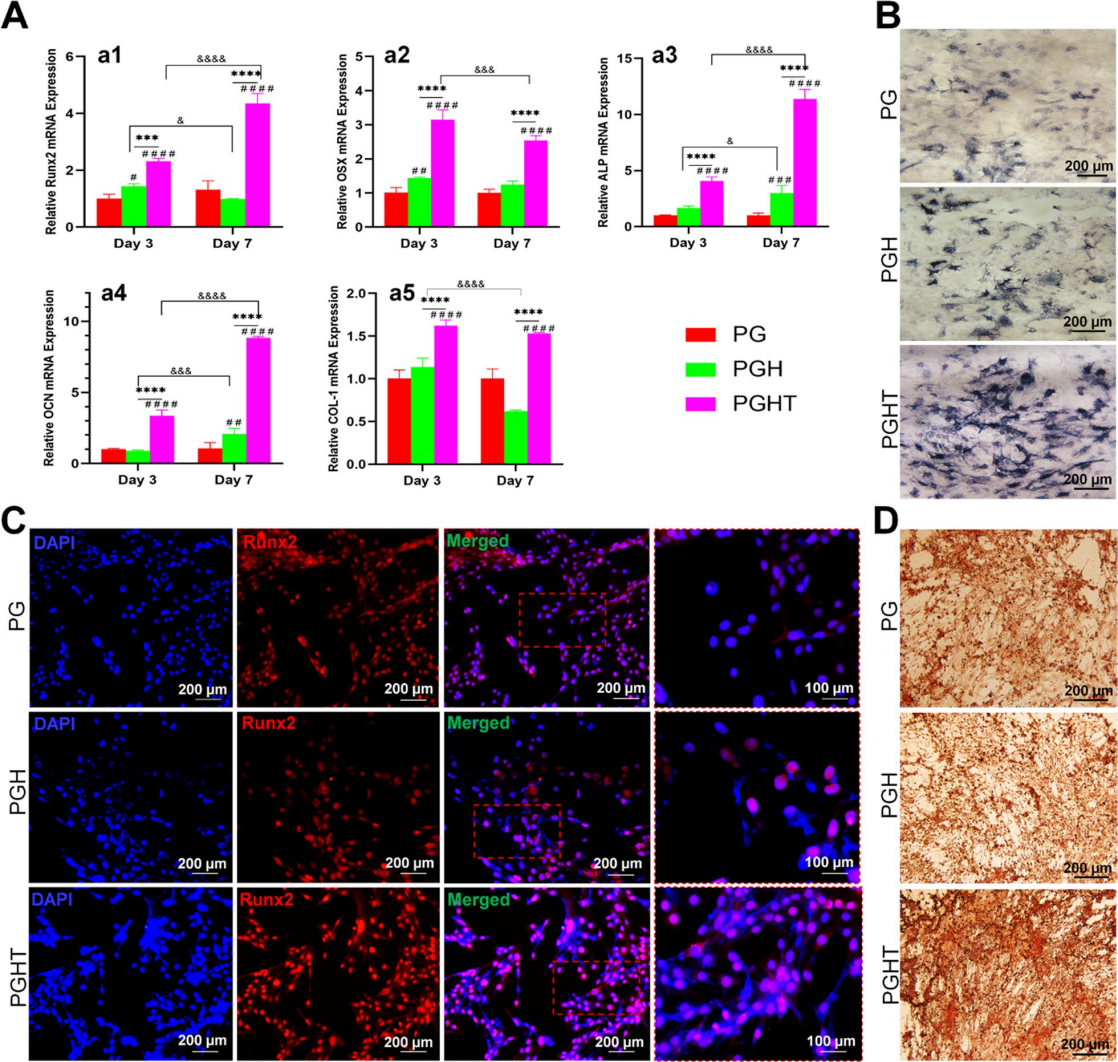
Osteogenic differentiation of D1 cells on the different membranes. **A** Expression of osteogenic marker genes: (**a1**) *Runx2*, (**a2**) *Osterix*, (**a3**) *ALP*, (**a4**) *OCN*, and (**a5**) *COL1*. **B** ALP activity staining of D1 cells on day 7. **C** Immunofluorescent staining of Runx2 expression on day 7 (blue, cell nucleus; red, Runx2). **D** ARS staining of D1 cells on day 21. ****p < 0.001; *****p < 0.0001 vs the PG membrane; ^###^*p* < 0.001. ^####^*p* < 0.0001 vs the PG membrane and the PGH membrane; ^&&&^*p* < 0.001. ^&&&&^*p* < 0.0001 vs day 3

To evaluate the osteogenic induction ability of the membranes, osteogenic-related gene expression levels were quantified using qPCR after D1 cells were cultured on the different membranes on days 3 and 7. *Runx2, OSX, ALP*, *OCN*, and *COL1* expression were elevated in the PGHT membrane in comparison with that of the PG and PGH membranes on days 3 and 7 (Fig. 6A). Additionally, the expression levels of *Runx2, ALP*, and *OCN* were markedly upregulated in the PGHT membrane on day 7 as compared to the PGH and PG membranes on day 3 (Fig. 6Aa1–a5). At the same time, the PGHT membrane exhibited significantly higher *OSX* and *COL1* levels compared to the PG and PGH membranes on day 7 (Fig. 6A-a2, a5). Immunofluorescent staining showed that the protein level of Runx2 was also increased in the PGHT membrane on day 7 in comparison with the PGH and PG membranes (Fig. 6B), indicating the PGHT membrane was more effective for the osteoblastic differentiation of BMSCs.

ALP staining of cells was greater with the PGHT membrane after incubation for 7 days than that with the PGH and PG membranes (Fig. 6C). Additionally, on day 21, Alizarin Red staining was performed to detect calcium nodules. More mineralized nodules were formed on the PGHT membrane, whereas minimal calcium nodule formation was observed on the PG and PGH membranes (Fig. 6D). ALP and Alizarin Red staining revealed that the PGHT membranes increased the ALP activity and calcium deposition.

#### In vitro angiogenesis evaluation

**Fig. 7:**
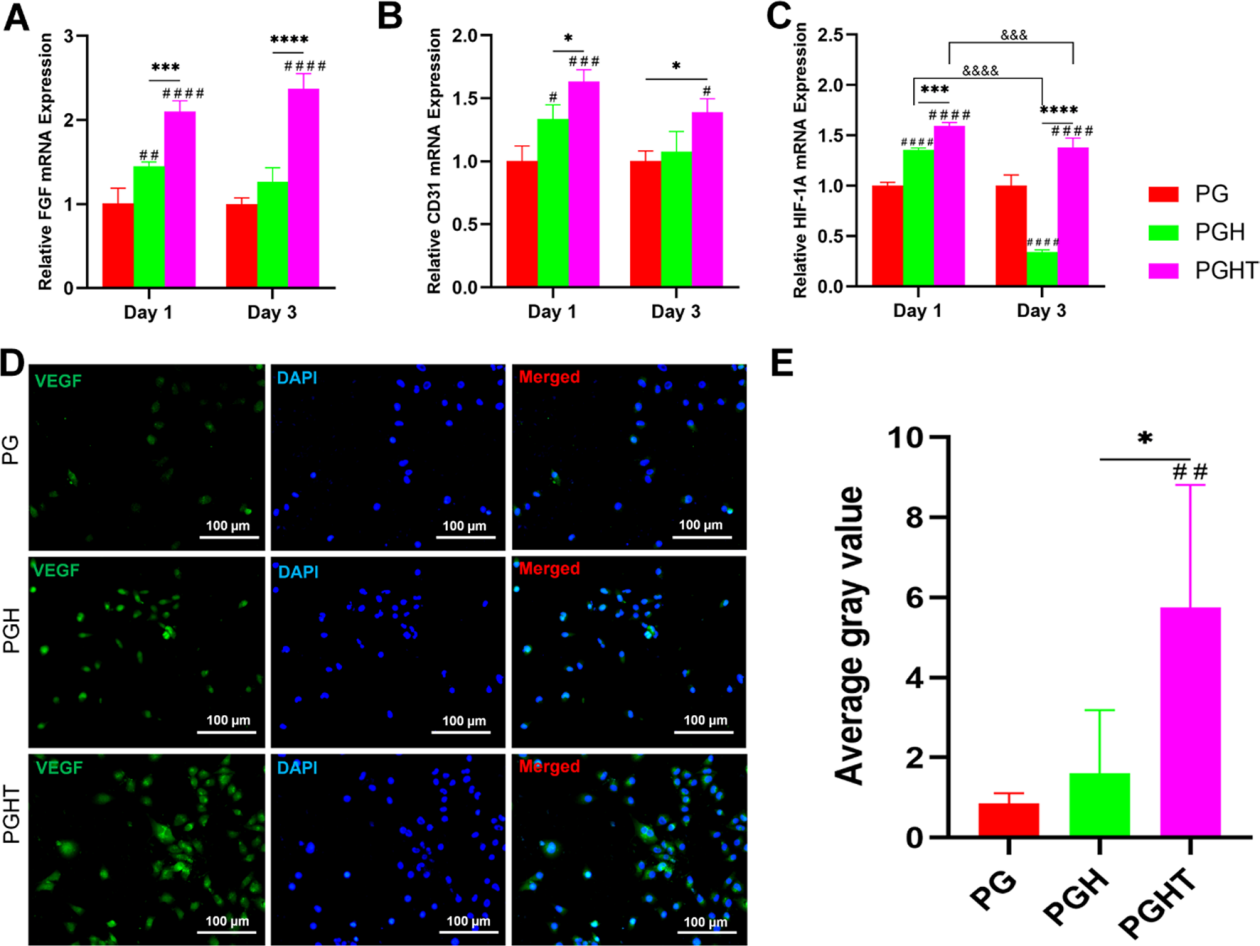
Vascularization of HUVECs on the different membranes in vitro. **A** FGF mRNA expression. **B** *CD31* mRNA expression. **C** *HIF-1A* mRNA expression. **D** Immunofluorescent staining of VEGF expression at 7 days (green, VEGF; blue, cell nucleus). **E** Semiquantitative data of VEGF expression from (D) (n = 3). *p < 0.05 vs PG membrane; *p < 0.05; ***p < 0.01 vs PGA membrane. ****p < 0.0001 vs PG membrane; ^####^*p* < 0.0001 vs PGH membrane; ^&&&^ *p* < 0.01; ^&&&&^ *p* < 0.0001 vs on day 3

To assess the ability of the different membranes for angiogenesis, HUVECs were co-cultured with the PG, PGH, and PGHT membranes for 1 and 3 days. qPCR showed that the expression of angiogenesis-related genes, such as *HIF-1A*, *FGF*, and *CD31*, was noticeably upregulated in the PGHT membrane compared with that of the PG and PGH membrane on days 3 and 7. Furthermore, the expression of these genes on the PGH membrane was enhanced as compared to the PG membrane (Fig. 7A–C).

To investigate the angiogenesis ability of the different membranes, immunofluorescence staining was used to evaluate the expression of VEGF. In the PGHT membrane, VEGF expression levels exhibited significantly higher at 3 and 7 days than that of the PG and PGH membranes (Fig. 7D). Furthermore, in the PGH membrane, the expression of VEGF was increased in comparison with that of the PG membrane (Fig. 7E). These results indicated that the PGHT nanofibrous membrane exhibited a favorable angiogenesis ability.

#### Micro-CT

**Fig. 8:**
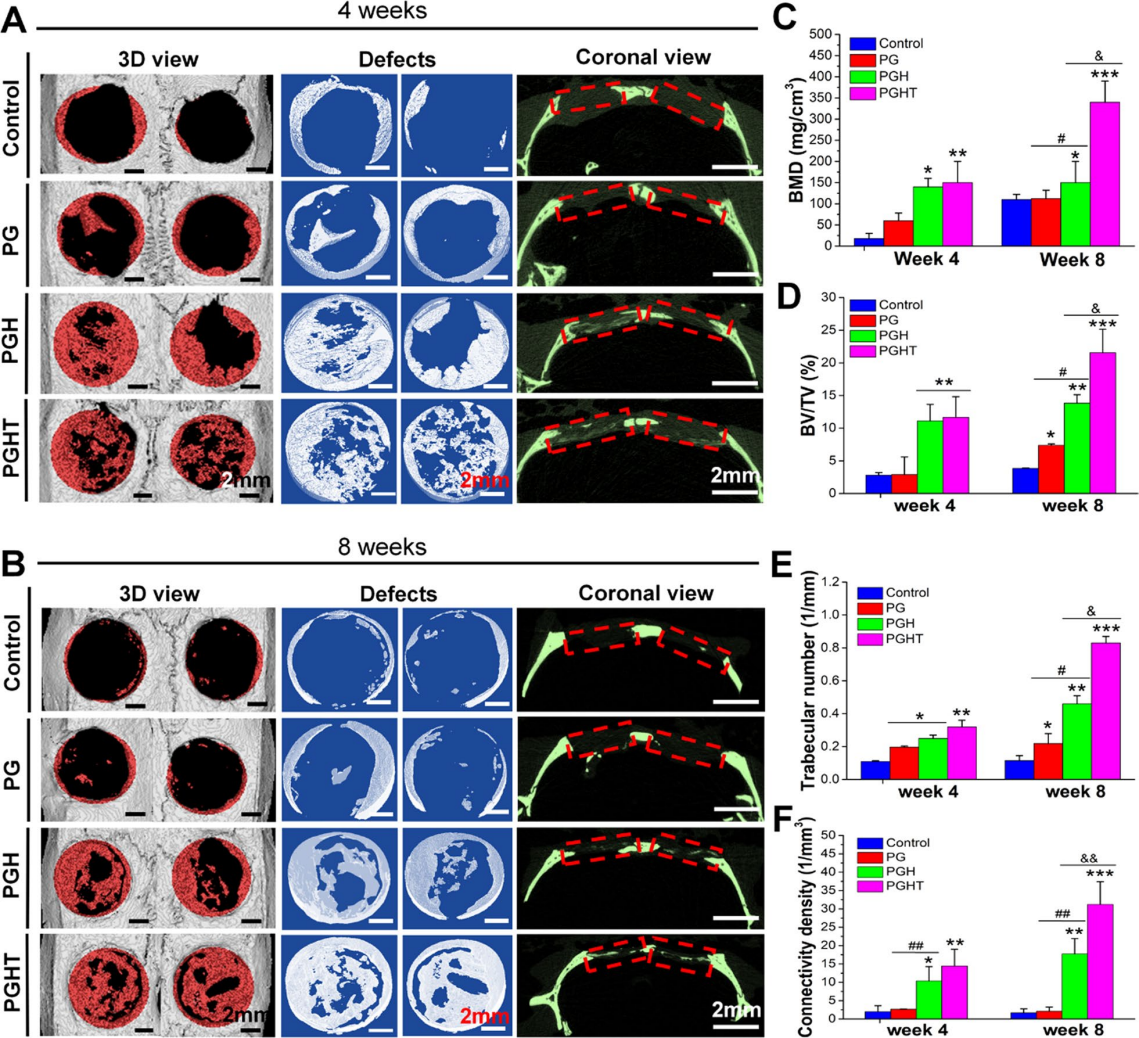
New bone regeneration enhanced by PGHT membrane in a rat cranial defect model. **A**, **B** micro-CT scan images at 4 (**A**) and 8 (**B**) weeks post-surgery. The newly formed bone tissue is marked in red within the 5-mm diameter circle. **C** Bone mineral density (BMD). **D** Percent bone volume (BV/TV). **E** Trabecular number. **F** Connectivity density (Conn. Dn) (n = 8). *p < 0.05, **p < 0.01, ***p < 0.001, vs. defect alone with no implanted materials; ^#^*p* < 0.05, ^##^*p* < 0.01, vs. PG membrane; ^&^*p* < 0.05, ^&&^*p* < 0.01, vs. PGH membrane

Micro-CT scans showed that new bone was not present in the control group at 4 and 8 weeks (Fig. 8A, B). Furthermore, only a small amount of newly formed bone could be seen in the PG group at 8 weeks post-surgery. However, we observed new bones in the PGH and PGHT groups. In particularly, in the PGHT group, a larger amount of the newly formed bone was present at week 8. The quantitative analysis of micro-CT provided evidence that the PGHT group had significantly larger values of BMD (104.5 ± 49 and 337 ± 52 mg/cm^3^ at 4 and 8 weeks, respectively), BV/TV (11.7 ± 3% and 21.6 ± 3.6% at 4 and 8 weeks, respectively), trabecular number (0.32 ± 0.04 and 0.83 ± 0.04 1/mm at 4 and 8 weeks, respectively), and connectivity density (14.1 ± 4.6 and 32.2 ± 6.2 1/mm^3^ at 4 and 8 weeks, respectively) compared with that of other groups, indicating TMP-loaded could increase cranial bone regeneration in rats (Fig. 8C-F)**.**

#### Histological analysis

**Fig. 9:**
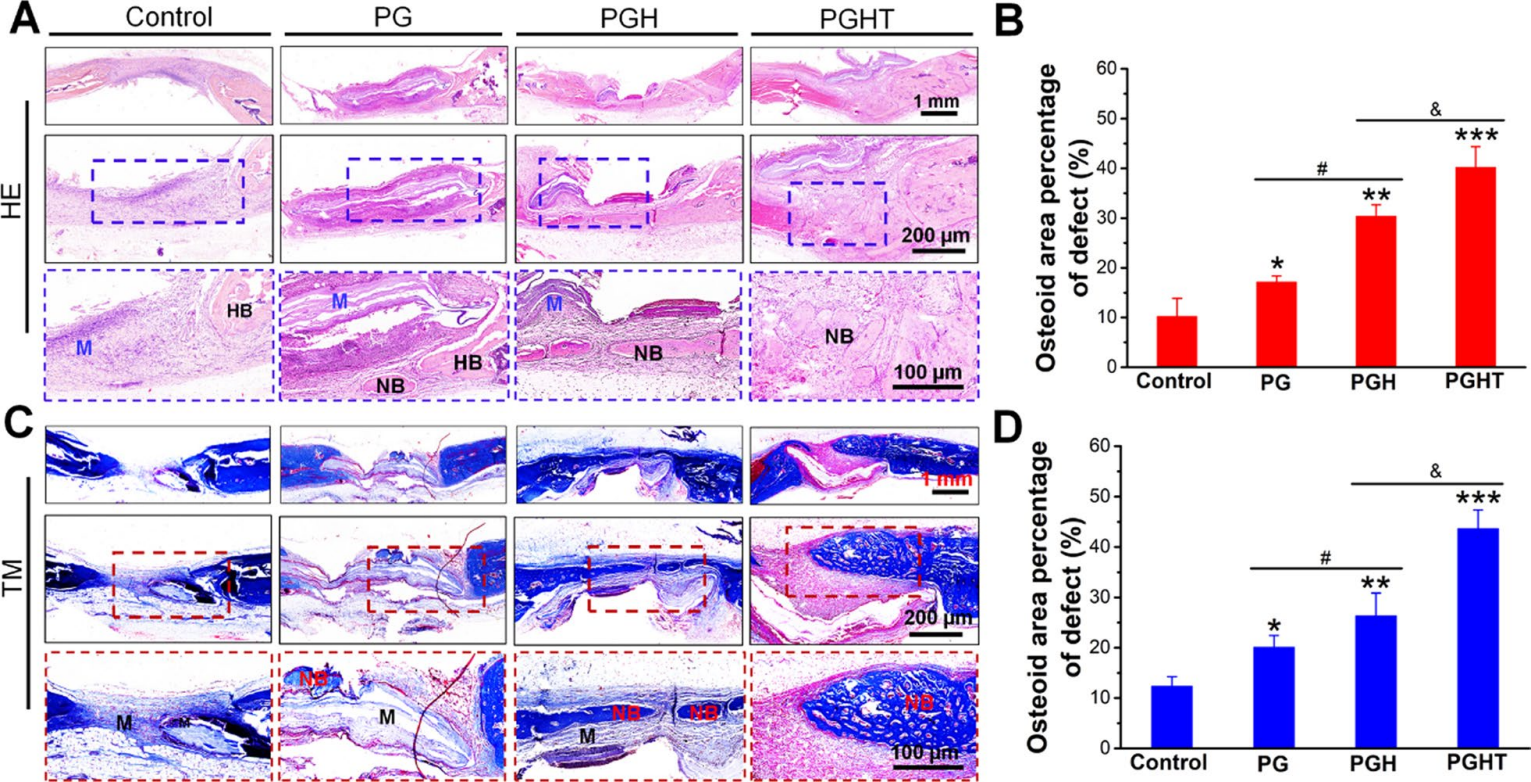
Histological staining results for the different membranes in the rat cranial defect model at 8 weeks post-surgery. **A** H&E staining images of regenerated tissue. **B** The quantitative analysis of H&E staining. **C** Masson’s trichrome staining images of regenerated tissue. **D** The quantitative analysis of Masson’s trichrome staining. * *p* < 0.05, ** *p* < 0.01, *** *p* < 0.001, vs. defect alone with no implanted materials group; ^#^ *p* < 0.05, vs. PG group; ^&^* p* < 0.05, vs. PGH group. NB: new bone. M: materials. HB: host bone

To verify the bone regeneration effect in the various groups, we performed H&E and Masson’s trichrome staining to evaluate bone regeneration. On week 8 post- surgery in the control group, a larger number of connective tissues was found in the bone defect area, although no obvious new bone was formed (Fig. 9A). Meanwhile, on week 8 post-surgery, only a few new bones were formed in the PG group. Conversely, we found clear evidence of a large amount of newly formed bone in the PGH group, and particularly in the PGHT group as compared to the other groups (Fig. 9B). Masson staining images at 8 weeks post-surgery showed that the collagen deposition in the control group was lower than that in the other groups. Additionally, in the PG group, the intensity of blue-stained collagen matrix was inferior to that of the control group. Conversely, the collagen deposition was higher in the PGH and PGHT groups, and a large amount of osteoid was observed as blue stains in the PGH and PGHT group (Fig. 9C). The PGHT group had a much higher intensity in Masson staining than that of the PGH group at week 8 post-surgery, which was consistent with the H and E results (Fig. 9D), indicating that PGH and PGHT increased the formation of new bone because of the deposition of HA and the role of TMP angiogenesis on the surface of the membranes.

#### Immunohistochemical evaluation of bone regeneration

**Fig. 10:**
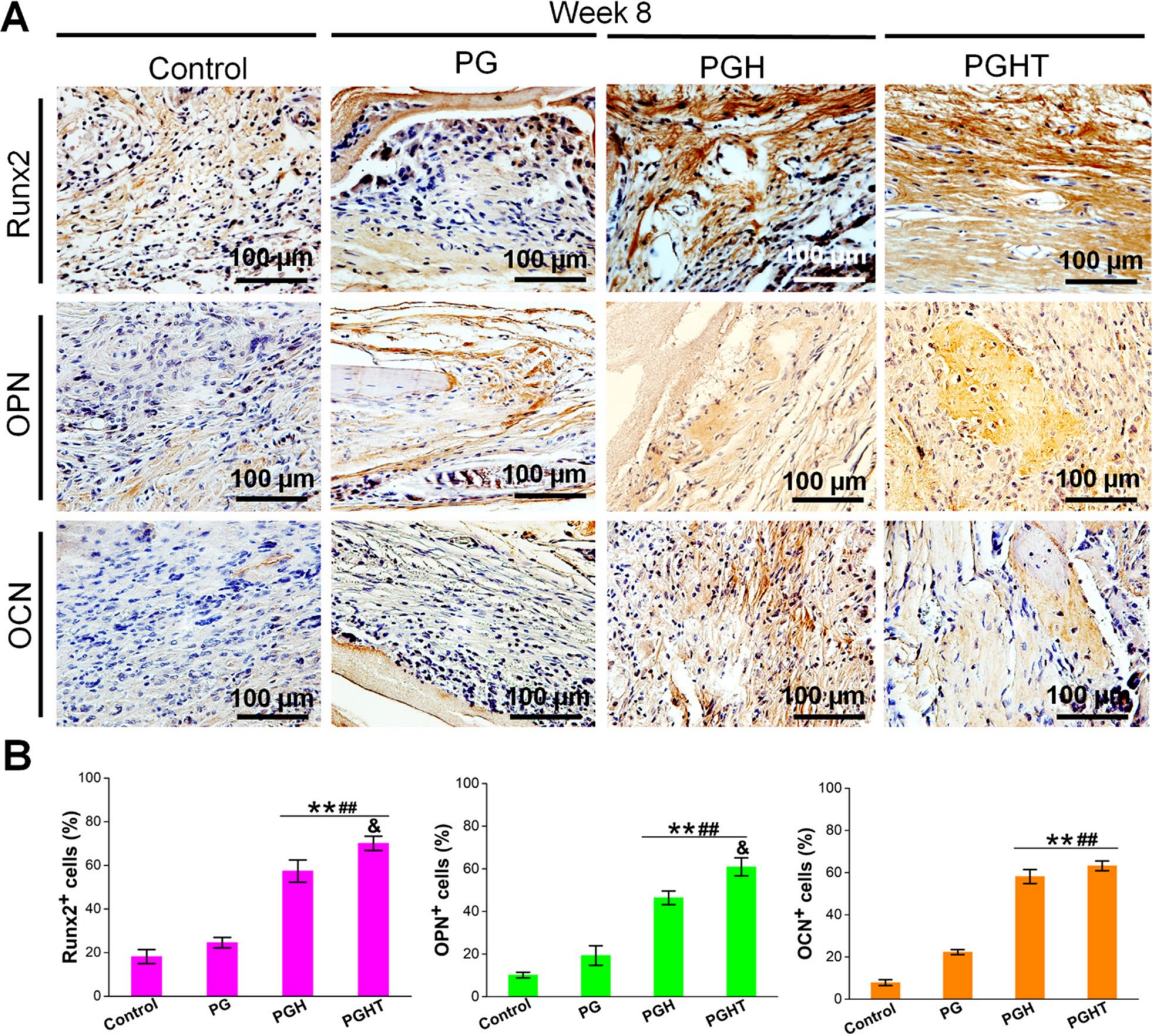
Immunohistochemistry staining results for different membranes in a rat cranial defect model at 8 weeks post-surgery. **A** Representative immunohistochemistry (IHC) staining images of Runx2, OPN, and OCN. **B** Semiquantitative analyses of IHC staining results from (A) (n = 8). * *p* < 0.05, ** *p* < 0.01, vs. defect alone with no implanted materials; ^##^ *p* < 0.01, vs. PG membrane; ^&^* p* < 0.05, vs. PGH membrane

Based on immunohistochemical staining findings for different membranes at 8 weeks post-surgery (Fig. 10), the levels of Runx2, OCN, and OPN, were noticeably higher in the PGHT group than that of the PGH and PG groups. Additionally, in the PGH group, the expression of Runx2 protein was higher than that of the PG group (Fig. 10A, B). Furthermore, OPN expression in the PGH group was higher than that of the PG and control groups, indicating that the PGHT membrane could promote osteogenic-related protein expression.

#### In vitro evaluation of the angiogenesis of the different membranes

**Fig. 11:**
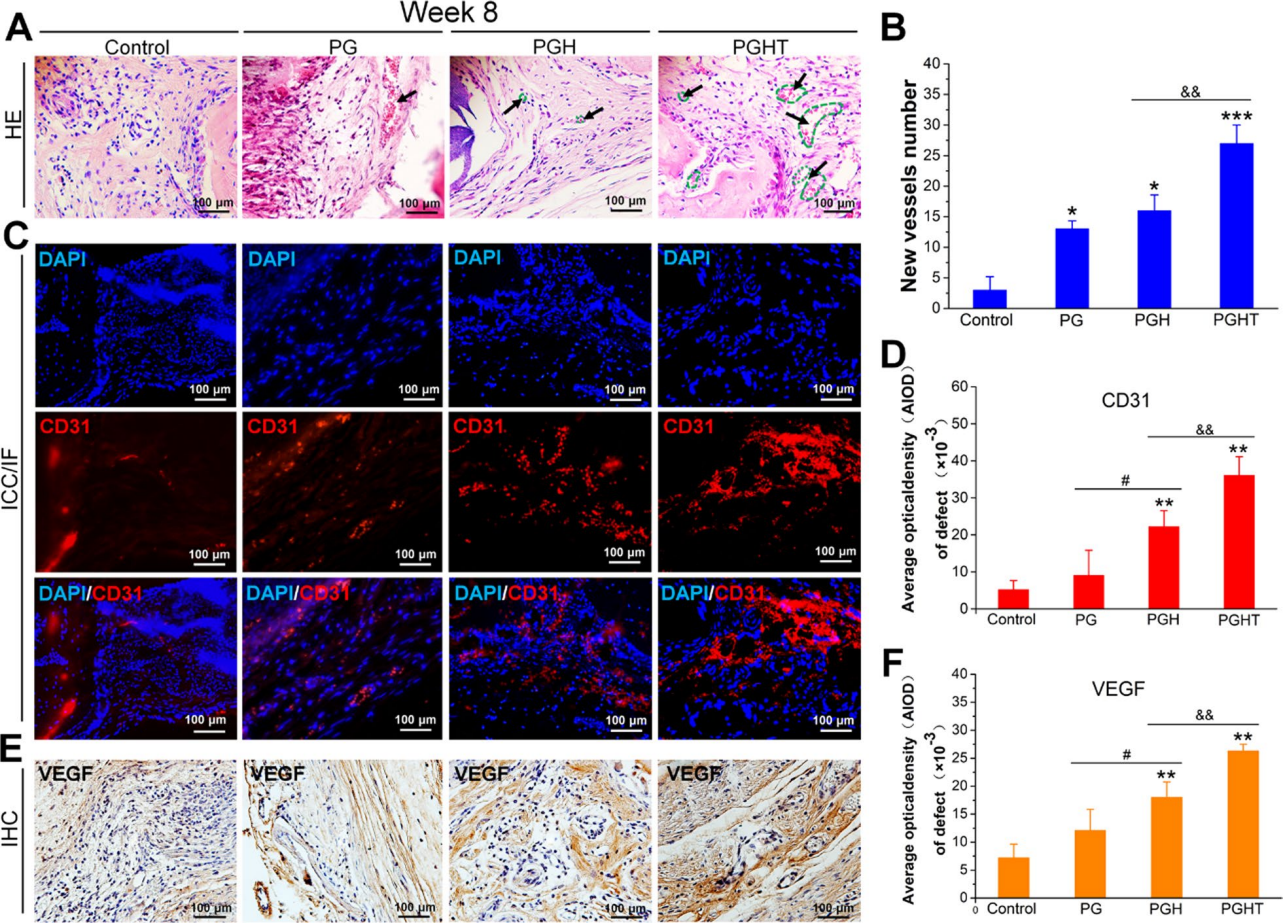
In vitro evaluation of the angiogenesis of different membranes in a rat cranial defect model at 8 weeks post-operation. **A** H&E staining images. **A** black arrow indicates new blood vessels. **B** Representative new vessel number from **A**. **C** CD31 immunofluorescence staining images; cell nuclei were labeled with DAPI (blue fluorescence), and CD31 protein was labeled with red fluorescence. **D** Quantitative analyses of CD31 expression from **C** in the different groups. **E** VEGF immunohistochemistry staining images. **F** Quantitative analyses of VEGF expression from **C** in the different groups (n = 8). * *p* < 0.05, **p < 0.01, ***p < 0.001, vs. defect alone with no implanted materials; ^#^*p* < 0.05, ^##^*p* < 0.01, vs. PG membrane; ^&&^*p* < 0.05, vs. PGH membrane

Angiogenesis was also assessed using H&E and immunofluorescence staining. In the PGHT group, the number of new blood vessels was notably enhanced compared with that of the other groups at week 8 post-surgery (Fig. 11). Furthermore, a higher number of new blood vessels were present in the PGH group than that in the PG and control groups at week 8 post-surgery (Fig. 11A, B). Immunofluorescent staining was performed to evaluate the expression of CD31, a biomarker of new vessels. The number of CD31-positive cells in the PGHT group significantly increased at 8 weeks post-surgery. Additionally, the PGH group exhibited a much higher expression of CD31 compared with that of the PG and control groups, the semiquantitative analysis of fluorescence in the PGH group was coincident with CD31 expression on week 8 post-surgery (Fig. 11 C, D). Subsequently, VEGF expression was assessed by immunohistochemistry staining at week 8 post-surgery (Fig. 11E). The PGHT group contained more patches of brown-stained areas than were present in the other groups. Furthermore, the PGH group contained more positive-stained areas than those in the PG group, and few positive-staining areas were present in the PG and control groups (Fig. 11E, F). Collectively, these results indicate that the PGHT membrane could increase vascularization, which contributed to bone regeneration.

## Discussion

Herein, we designed and developed a novel biomineralized TMP-loaded PCL/Gelatin nanofiber membrane containing bubble-like forms using electrospinning technology for bone tissue engineering that incorporated (1) a mixture of PCL, gelatin, and paraffin to form electrospun membranes; (2) washing of the membranes with cyclohexane to remove paraffin formed bubble-shaped nanofibers; (3) Biomineralization achieve TMP-loading on the surface of the nanofiber membranes. Our objective was to overcome the disadvantage of conventional electrospinning that produces a smooth nanofiber surface that limits the ability of the biomineralization. Our results show that the surface of the electrospun nanofiber exhibited the bubble-shaped structural features when the nanofiber membranes were washed with hexane and cyclohexane to remove the paraffin spheres. We use this approach to add nanofibers with the following characteristics: (1) The prepared paraffin microspheres can form bubble like structures of varying sizes on the surface of PCL nanofibers, which can effectively increase the surface roughness of nanofibers compared to sodium chloride or other inorganic materials loaded (such as hydroxyapatite, nano-clay, etc.), which is promote to biomineralization and HA formation. (2). The formation of the bubble-like structures of varying sizes on the surface of nanofibers increases the specific surface area of the fibers, which is conducive to the formation of a large amount of crystal HA and drug loading. Our results also confirm that the foam like PCL/Gelatin nanofiber membrane formed by loading paraffin microspheres can significantly promote biomineralization compared to the PG nanofiber membrane material. Therefore, this approach as a novel strategy to increase the surface roughness of the electrospun nanofiber.

Biomineralization can reflect the ability of the membrane to form apatite crystal deposits on its surface. Accordingly, in this study, we successfully fabricated the biomineralized TMP-loaded PG nanofibers membranes. The ability of biomineralization in the PG membrane doped with paraffin spheres was higher than that of the PG membrane that was not doped with them. The main reason was that the paraffin spheres formed bubble-like shapes on the nanofibers that increased the surface area and roughness of the electrospun nanofibers and consequently increased the deposition of calcium phosphate. Additionally, compared with the PG membrane, the PGH membranes exhibited superior mechanical characteristics in terms of the elongation at the break modulus. This could be explained by the mineralization of PG membrane with nano-HA on the surface subsequently enhanced the mechanical properties. Our results are similar to those of previous reports. Russias et al. fabricated PLA-based composites with 70–85 wt% mHA that exhibited mechanical properties that closely match those of human cortical bones [38]. Nie. et al. prepared a hierarchical PLLA/PCL nanofibrous scaffold using a one-pot thermally induced phase separation that was then rapidly mineralized by an electrochemical deposition technique. Here, the ultimate strain in the mineralized-PLLA/PCL was markedly enhanced in comparison with that of PLLA/PCL only, indicating that the mechanical properties of membrane materials are increased after mineralization [39].

In tissue engineering, controlled release of drugs from materials is central to tissue repair and regeneration [40, 41]. The controlled release of drugs contributes to establishing suitable localized drug concentrations; meanwhile, drugs must meet a minimum threshold without causing side effects to cells and tissue. Various materials, such as natural and synthetic materials [42, 43], microspheres [44, 45], hydrogels [46, 47], nanofibrous [48, 49], silica aerogels [50], and composite material systems [51, 52], can be used as the drug release system in tissue engineering. Therefore, a new approach to improving the controlled release of drugs for tissue engineering is urgently required. Coaxial electrospinning is an effective strategy to achieve the controlled release of drugs for an extended period [53, 54]. However, the coaxial electrospinning scaffolds are not easy to successfully fabricate for the controlled release of drugs. Additionally, although the drug is directly loaded on the surface of nanofiber membranes to realize the drug delivery, the controlled release may not meet the demand for tissue regeneration. Biomimetic mineral deposition where drugs are loaded into crystalline HA on scaffolds is an ideal approach for the controlled release of drugs [33]. Herein, TMP was loaded on the PG membrane to use the mineralization of the HA approach. The controlled release of the drug showed a burst-release of 39.8 ± 1.4% TMP were released within 2 days. Furthermore, the cumulative release of TMP was 61.0 ± 1.8% at day 28, indicating our approach of loading drug on nanofiber membrane had an excellent drug release efficiency. The results of this study confirmed that the use of biomineralization methods to load ligustrazine can achieve sustained release of the drug, due to the fact that, firstly, ligustrazine has two hydrogen bonding receptor sites and is prone to forming hydrogen bonds with -OH. The nano hydroxyapatite (rich in -OH) formed during the biomineralization process can bind with ligustrazine through hydrogen bonding, achieving drug loading and sustained release [55]. Secondly, the nano hydroxyapatite formed on the surface of the fiber membrane through biomineralization has a large specific surface area, which can not only bind drugs through hydrogen bonding, but also locally re-adsorb released drugs, achieving sustained release function through drug gradient [56]. Finally, the nano hydroxyapatite on the surface of the fiber membrane can promote bone regeneration, while the drug loaded hydroxyapatite can integrate into the newly formed bone tissue, delaying the drug release rate [57].

Vascularization is recognized as being critical for the formation of bones [58–60]. In our study, we found that the PGHT membrane could upregulate the expression of the angiogenic-related genes and increase the VEGF protein expression, enabling the PGHT membrane to effectively promote angiogenesis. This may be due to the loading of TMP in the biomineralized HA and its controlled release effect, which increased the expression of VEGF protein and consequently promoted the tubular formation of HUVECs. This is because VEGF is central to regulating angiogenesis [61, 62]. VEGF not only promotes endothelial cell migration and proliferation but also activates endothelial cells to form blood vessels [63].

To evaluate the effect of bone regeneration on the different membranes, a critical-sized defect in rat cranium was created. Micro-CT results showed that significant active bone regeneration occurred on the PGHT membrane. In vivo H&E and Masson’s trichrome staining data demonstrated that new bone had formed in the PGH and PGHT groups at 4 weeks of implantation. However, by 8 weeks, more newly regenerated bone was found in the cranium defect area in the PGHT group. By contrast, only a smaller amount of newly regenerated bone was found in the PG and control groups, indicating the PGHT membrane increased new bone formation and accelerated bone generation. PCL/Gelatin electrospun nanofiber has been widely reported as a bone graft recently. Ren et al. electrospun homogeneous PCL/Gelatin with different ratios to successfully fabricate nanofiber membranes that promoted bone formation in vitro [64]. Gong et al. reported that the traditional Chinese medicine icariin (ICA) was doped into the electrospun PCL/Gelatin nanofibers to fabricate an artificial periosteum. The ICA-loaded electrospun membranes induced significantly increased ALP activity and enhanced the expression of OCN and COL I in MC3T3-E1 cells [65]. Additionally, biomimetic nanofibrous materials have been widely used for bone regeneration. Wang et al. successfully fabricated biomimetic mineralized hydroxyapatite nanofibers with a methacrylic acid gelatin hydrogel to form a composite hydrogel for bone regeneration. This novel biomimetic mineralized hydroxyapatite nanofiber composite GelMA hydrogels enhanced bone regeneration [66]. Herein, our study also indicated that biomimetic mineralized PCL/Gelatin nanofibers have an excellent effective bone regenerative.

Vascularization is central to the development, maturation, remodeling, and regeneration of bone [67, 68]. Furthermore, the vascularization and bone regeneration have a close functional relationship; the more blood vessels formed, better the effect of bone regeneration and repair [23, 69]. Importantly, in this work, the biomineralized TMP-loaded PCL/Gelatin nanofibers significantly promoted blood vessel formation. H&E and immunofluorescence staining demonstrated a significantly increase in the number of new blood vessels in the PGHT membrane at 8 weeks. Moreover, CD31 and VEGF expression was notably increased with the PGHT membrane, indicating that biomineralized TMP-loaded PCL/Gelatin nanofibers can increase vascularization because of the use of the biomineralized PCL/Gelatin membrane loaded with TMP. Thus, TMP can promote the growth, migration, and vascularization of endothelial cells while the TMP and biomineralized HA synergistically enhanced on vascularization in rat cranium defects, indicating the excellent vascularized and bone formation effects of the biomineralized PG membrane loaded with TMP on local bone defects.

## Conclusion

Herein, we successfully fabricated a biomineralized TMP-loaded PCL/Gelatin shape-bubble nanofiber membranes using electrospinning technology and demonstrated their potential in bone tissue vascularization and regeneration. A PCL/Gelatin nanofiber had increased roughness of the nanofiber due to the presence of bubble-like shapes on the surface. Importantly, this novel nanofiber could improve the biomineralization and drug-loading ability. Furthermore, in vitro TMP-loaded PCL/Gelatin nanofiber membranes significantly increased cell attachment and proliferation, osteoblast differentiation, and vascularization. In vivo the nanofiber membranes promoted vascularization and bone formation in rat cranium defects. Overall, this novel biomineralized TMP-loaded PCL/Gelatin nanofiber membrane has strong potential for bone regeneration.

## Data Availability

Not applicable.
